# Clonal evolution of acute myeloid leukemia highlighted by latest genome sequencing studies

**DOI:** 10.18632/oncotarget.10850

**Published:** 2016-07-26

**Authors:** Xuehong Zhang, Dekang Lv, Yu Zhang, Quentin Liu, Zhiguang Li

**Affiliations:** ^1^ Center of Genome and Personalized Medicine, Institute of Cancer Stem Cell, Cancer Center, Dalian Medical University, Dalian, China; ^2^ State Key Laboratory of Oncology in South China, Cancer Center, Sun Yat-sen University, Guangzhou, China; ^3^ Department of Hematology, The Third Affiliated Hospital, Sun Yat-sen University, Guangzhou, China; ^4^ Institute of Hematology, Sun Yat-sen University, Guangzhou, China

**Keywords:** acute myeloid leukemia, clonal evolution, cancer genome

## Abstract

Decades of years might be required for an initiated cell to become a fully-pledged, metastasized tumor. DNA mutations are accumulated during this process including background mutations that emerge scholastically, as well as driver mutations that selectively occur in a handful of cancer genes and confer the cell a growth advantage over its neighbors. A clone of tumor cells could be superseded by another clone that acquires new mutations and grows more aggressively. Tumor evolutional patterns have been studied for years using conventional approaches that focus on the investigation of a single or a couple of genes. Latest deep sequencing technology enables a global view of tumor evolution by deciphering almost all genome aberrations in a tumor. Tumor clones and the fate of each clone during tumor evolution can be depicted with the help of the concept of variant allele frequency. Here, we summarize the new insights of cancer evolutional progression in acute myeloid leukemia.

Cancer evolution is currently thought to start from a clone that has accumulated the requisite somatically-acquired genetic aberrations through a series of increasingly disordered clinical and pathological phases, eventually leading to malignant transformation [1–3]. The observations in invasive colorectal cancer that usually emerges from an antecedent benign adenomatous polyp and in cervical cancer that proceeds through intraepithelial neoplasia support the idea of stepwise or linear cancerous progression [3–5]. Genetically, such progression is achieved by successive waves of clonal expansion during which cells acquire novel genomic alterations including single nucleotide variants (SNVs), small insertions and deletions (indels), and/or copy number variations (CNVs) [6]. The latest improvement in sequencing technology has allowed the deciphering of the whole exome or genome in different types of tumor and normal tissue pairs, providing detailed catalogue about genome aberrations during tumor initiation and progression, which have been reviewed in several papers [7–10]. Here, we focus on demonstrating the cancer clonal evolution pattern revealed by recent deep sequencing studies of samples from acute myeloid leukamia (AML) patients.

## CLONAL EVOLUTION IN AML PATIENTS

**Figure 1:**
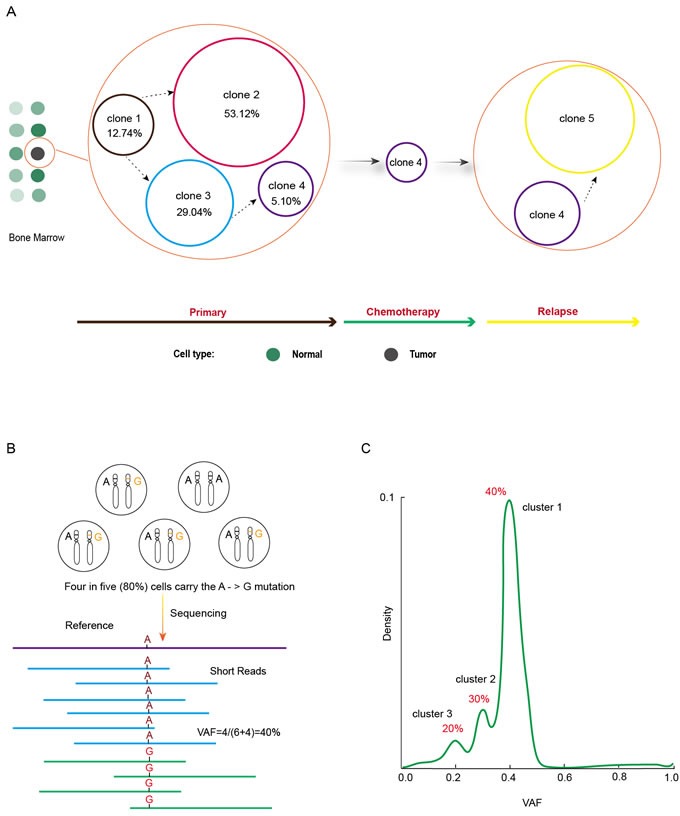
Clonal evolution revealed by cancer genome studies **A.** Five distinct clones successively emerged in an AML patient with clone 4 surviving chemotherapy and evolving into clone 5 by the acquisition of novel drug-resistant mutations. **B.** Four of five dipoid tumor cells harbor the variant nucleotide of Guanine on one of the two homogenous chromosomes at the position of reference nucleotide of Adenine. Current sequencing technology does not discriminate the homogenous chromosomes and results in 10 short reads with 4 of them carrying variant nucleotide of G and 6 carrying reference nucleotide of A. Therefore, the variant allele frequency (VAF), 40% here, is equal to half of the percentage of mutation-carrying tumor cells, which is 80%. **C.** Tumor clones can be detected from the density plot of VAF with each peak representing a clone that carries the mutations defined in that peak.

To study the evolutional course of cancer genome in AML patients, investigators performed whole-genome sequencing of primary tumor, relapse tumor and matched skin samples from eight patients [11]. As expected, they found somatic mutations in known AML genes such as DNMT3A, FLT3, NPM1, IDH1, IDH2, WT1, RUNX1, PTPRT, PHF6 and ETV6, as demonstrated in several other studies [12–22]. Most importantly, major clonal evolution patterns during AML relapse were demonstrated as the founding clone or a subclone of the founding one survived initial therapy, gained additional mutations and expanded at relapse [11]. To elucidate somatic mutation changes between primary and relapsed tumor genome, we made a schematic diagram according to the data in one of the patients (Figure 1A). Four clones numbered 1 to 4 were present in primary tumor at the percentage of 12.74%, 53.12%, 29.04% and 5.1% respectively in this patient. Clone 2 and 3 were evolved from clone 1 and included all somatic mutations in clone 1. Clone 2 either appeared earlier than clone 3 or grew more rapidly than clone 3 since this clone comprised more proportion of tumor cells. It is likely that a small portion of clone 3 cells acquired new genomic variants to form clone 4. This should be a late event in the evolution path because clone 4 only comprised 5.1% tumor cells. With the onset of chemical treatment, all cells would face the fate of either dying out or changing to acquire novel drug-resistant mutations. It turned out clone 1, 2, and 3 cells totally succumb to chemical therapy. Most cells in clone 4 were also killed during the therapy by the combined treatment of drugs of cytarabine, daunorubicin, and etoposide, mitoxantrone, cytarabine, and fludarabine, as well as interleukine 12 (IL-12) [11]. However, relying on the 78 somatic alterations that are either preexisted or newly acquired, a subset of clone 4 cells finally evolved into a new clone, clone 5, which seemed to have the capability to resist all the treatments and eventually led to the expiration of the patient. The dynamics and plasticity of cancer genome is clearly illustrated by the supersession of different tumor cell clones in this patient (Figure 1A). Similar clonal evolution was also observed in other seven patients [11]. Tumor clonal architecture is prevalent in AML. Clonality analysis of whole-genome sequencing (WGS) data from 50 AML patients found more than half the tumors contained both a founding clone and at least one subclone; five patients had two subclones and as many as three independent subclones were identified in one patient [23].

Clonal evolution was also reported during the progression from the myelodysplastic syndromes (MDS) to secondary AML (sAML) [24]. Specimens of MDS, sAML, and matched skin were obtained from seven patients and subjected to WGS analysis and the findings of SNVs, indels, and CNVs were validated by custom solid-phase long-oligonucleotide arrays [24]. In one of the patients, five mutation clusters were identified according to variant allele frequency (VAF) of SNVs in MDS and sAML. In addition, CNVs were also subject to clustering analysis and three distinctive clusters were discerned, with two of CNV clusters presumably corresponding to two SNV clusters, increasing the credibility of clustering methodology for clonal identification. In total, five samples at MDS stage (averagely 2.4 clones per patient) and all seven samples at sAML stage (averagely 3.1 clones per patient) were multiclonal [24]. Clonal evolution was also detected in other types of sAML. In a patient experiencing the transformation from primary myolofibrosis (PMF) to sAML, unsupervised clustering of VAFs identified three clones at each of the three stages of PMF, sAML, and relapsed sAML. PMF-dominant clone was driven predominantly by JAK2 and U2AF1 mutations, which was successively superseded by the clone harboring ASXL1 and HCFC1 mutations, and then by the clone harboring RUNX1 and IDH1 mutations [25].

In addition, clonal architecture and evolution analysis is applicable to solid cancers. Using a VAF-based reconstruction method, it was found that subclonal diversity in 55 small cell lung cancers was threefold lower than 11 lung adenocarcinomas [26, 27]. A similar notion using allele frequency of germline SNP data is useful to deconvolve the sequence of gene deletions that give rise to malignant tumors. Such analysis revealed a “consensus path” of prostate tumor progression from the WGS data of 57 patients. Tumor progression began with events including the deletion of NKX3-1 or FOXP1 and fusion of TMPRSS2 and ERG, which might disrupt normal prostate epithelial differentiation [28, 29]. Thereafter, lesions in CDKN1B or TP53 accumulated; these alterations might lead to enhanced proliferation, genomic instability and/or evasion of apoptosis. Finally, loss of PTEN provided a gating event in the development of aggressive prostate cancers [30].

## THE UNDERLYING PRINCIPLES FOR TUMOR CLONE IDENTIFICATION

The crucial question in studying clonal evolution during cancer progression is how to define clones, such as the number of clones and what mutations each clone carries, in a tumor. Variant Allele Frequency (also called mutant allele frequency), defined as variant read count / (variant read count + wild type read count), is a very useful concept here [22]. A basic assumption in deep sequencing-based cancer genome studies is that somatic variants are heterozygous in tumor cells. Of course it cannot be excluded that a few tumor cells happen to be mutated at the same position to the same variant nucleotide on the two alleles, but the chances should be low [11, 22, 24]. Given the fact that each cell has two sets of chromosomes and a read from whole genome sequencing after removing PCR duplicates represents one chromosome, it can be deduced that the percentage of variant-carrying tumor cells is twice the VAF value, i.e., suppose 80% tumor cells harbor a mutation at a specific site, the VAF at this site should be 40% (Figure 1B). The number of clones and the variants harbored by each clone can be identified from the density plot of VAF. As shown in Figure 1C, three mutation clusters are discernable in a tumor sample with each cluster having VAF of 40%, 30%, and 20% respectively. Based on the idea represented in Figure 1B, these three clusters are supposed to exist in 80%, 60%, and 40% of tumor cells, which means that tumor cells carrying cluster 2 mutations should also carry cluster 1 mutations and the cells carrying cluster 3 mutations should simultaneously carry cluster 2 and 3 mutations as well. A clonal evolution course can be deduced that initial normal cells acquire cluster 1 mutations to start malignant transformation, and some cells gain cluster 2 mutations that might confer growth or survival advantages, and then a few cells obtain more mutations defined in cluster 3 to get even more malignant potentials such as inducing angiogenesis or metastasizing to distal sites. Tumor clones are then defined according to the mutation clusters [11].

**Figure 2:**
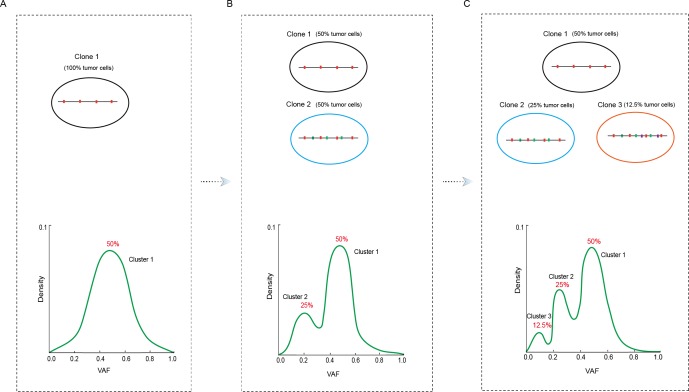
Schematic representation of the conversion between tumor clonal architecture and somatic mutation clusters **A.** Tumor cells in the initial population comprise the identical set of mutations shown in magenta bars; **B.** A subset of tumor cells obtains a new set of mutations (cyan bars) to form clone 2; and **C.** some of which further acquire one more set of mutations to form clone 3 (purple bars). Numbers in the parentheses denote the assumed percentage of tumor cells carrying the corresponding set of mutations at the sampling time. The low part in each panel shows the VAF density plots and mutation clusters that correspond to the clonal structures shown in the upper part.

To more clearly illustrate how the information of VAF clusters are converted to the hypothesis about genetic clonal cell populations, we made a schematic representation of tumor evolution during which novel mutations are progressively acquired (Figure 2). Initially, tumor population is relatively pure and a set of mutations are ubiquitously present in almost all tumor cells. The VAF of these mutations would follow a normal distribution centered at 50% (Figure 2A). With tumor progression, a new set of mutations arise at some time point, which leads to the emergence of a new clone that harbor both the original and newly acquired set of mutations (Figure 2B). Assuming half of tumor cells belong to the new clone at the time sampling for sequencing analysis, the VAF plot would have two peaks centered at 50% and 25% respectively. With further tumor progression, some tumor cells obtain the third set of mutations and form clone 3. Suppose 1/4 tumor cells have this set of mutations, the VAF plot would have three peaks centered at 12.5%, 25%, and 50% (Figure 2C). Practically, we need to infer clonal architecture from VAF density plot, such as a peak centered at 50% represents a tumor clone that only carry the set of mutations almost ubiquitously present in all tumor cells, while the peak centered at 25% represents a clone that, besides the ubiquitously present mutation set, also carry an additional set of mutations.

**Figure 3:**
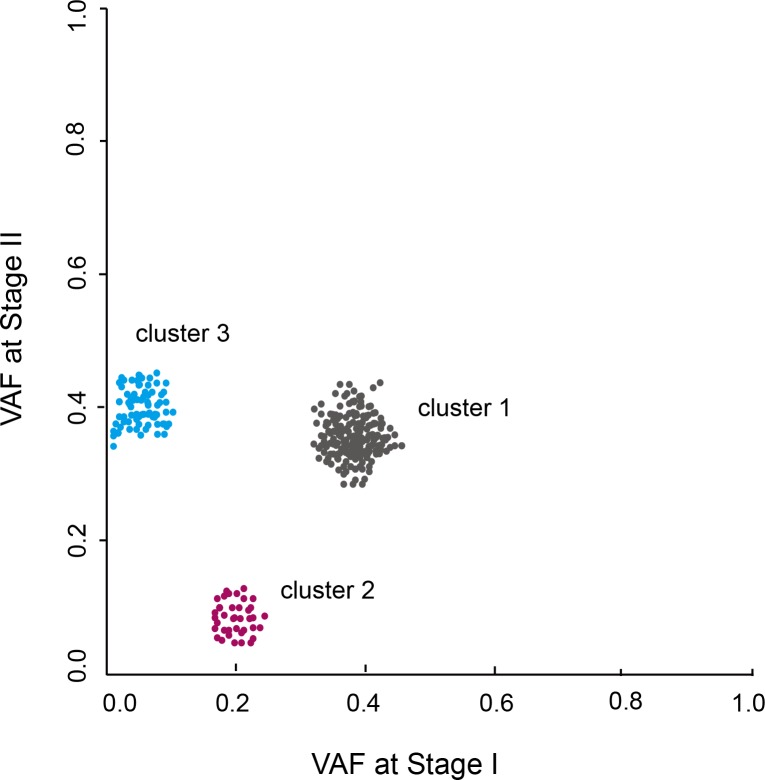
VAF clustering analysis of patients sequenced at two tumor stages Three clusters of mutations are identified. The changes of VAF represent the clonal evolution from stage I to II.

If two stages of sample, like primary and relapsed tumors, get sequenced, a scatter plot can be made between VAFs at the two stages to exhibit the progressive clone changes during the evolution from one stage to another. As shown in Figure 3, three mutation clusters are identified. Cluster 1 has VAF of 40% at both stages, indicating clones carrying this cluster of mutations keep stable during the progression from stage I to II. Cluster 2 has VAF of 20% in stage I but decreases to 0% at stage II, indicating this cluster of mutations cannot confer growth advantages to tumor cells or even restrain tumor growth that leads to the disappearance of cells harboring this cluster of mutations. In contrast, cluster 3 have VAF of 0% in stage I but increases to 40% at stage II, indicating this cluster of mutations are newly acquired and confer substantial growth advantages and leads to the cells with this cluster of mutations becoming dominant at stage II.

## HETEROZYGOSITY OF SOMATIC MUTATIONS IN TUMOR CELLS

**Figure 4:**
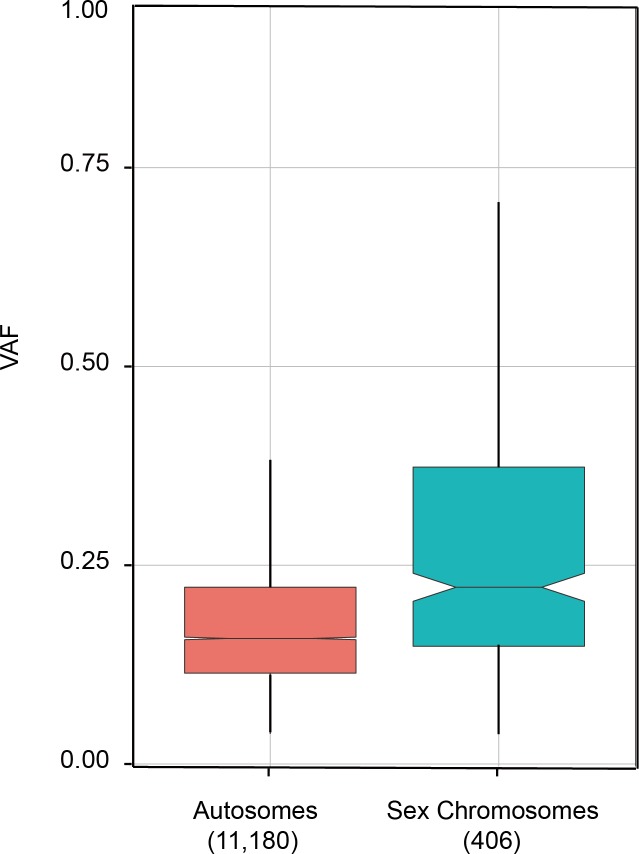
Comparison of VAFs on autosomal and sex chromosomes Whole genome sequencing was applied to tumor and peri-tumor tissues of a lung adenocarcinoma patient. VAFs were calculated for somatic mutations on autosomes (1-22 chromosomes) and sex chromosomes (X and Y chromosomes). Numbers in parenthesis are the number of somatic mutations detected on autosome and sex chromosomes. Sequencing was done on Illumina Hiseq platform with pair end 150 bp and overall depth reached 30x.

As stated above, one fundamental assumption for deducing cancer clone evolution from VAF is that somatic mutations emerge on only one allele on the two homologous chromosomes. Based on this assumption it can be inferred that a somatic event with VAF of 50% should be present in all tumor cells, and two events with VAF of 40% should be simultaneously present in the same set of 80% tumor cells. It is experimentally difficult to directly demonstrate this heterozygosity assumption except the statistical inference that chances are very low that two independent events emerge at the same position on two alleles with the same type of nucleotide substitution in one cell. However, the examination of VAF in copy number changed (gain or loss) genomic regions does provide some evidences. As shown in [31], most of copy neutral regions (diploid genome regions) have VAF of around 37%. However, at haploid genomic regions, such as the deletion of one chromosome 7 arm, regions of loss of heterozygosity at chromosome 16 and 20, X, or Y chromosome, the VAF value could reach 60% or even 80%. It is understandable that in the genomic regions with copy number loss, VAF could pass the limit of 50% if more than half tumor cells harbor mutations. In our whole genome sequencing study of lung adenocarcinoma, we also found that VAF of sex chromosomes (median=0.22) are higher than autosome chromosomes (median=0.16, Figure 4).

## BACKGROUND MUTATIONS THAT ARE NOT RELEVANT TO MALIGNANT TRANSFORMATION

It is now appreciated that tumor cells usually contain many somatic mutations that are not related to tumor initiation and progression. These mutations spread evenly across the whole genome, randomly acquired during cell growth, even prior to tumor initiation. They are just captured by the transformed cells and passed to all progenies during clonal expansion [31]. This conjecture stems from the observation that the VAF of mutations in cluster 1 (i.e., founding clone) usually reach almost 50%, which suggests all tumor cells carry such mutations. Moreover, the number of mutations detected by whole genome sequencing is usually far more than the number that is supposed to be biologically relevant. Around 440 somatic events were identified in 12 M1 and 12 M3 AML patients [31]. But most of them are not shared across the patients. Only 4-5 of these events recurrently occurred in the 24 patients and in a larger cohort study involving 200 AML patients [23]. A study involving whole exome sequencing of hematopoietic stem/progenitor cells from seven healthy individuals of different ages confirmed the idea that normal human cells randomly accumulate mutations over time [31]. It was found that the number of mutations was lowest in the cord blood samples and increased as a function of age; in adult volunteers, the number of mutations detected in each exome of healthy individuals was similar to that detected in AML patients of the same age; the mutational spectrum was very similar to that of AML samples with predominant transitions from C to T, suggesting a role of deamination of methylcytosine residues [31]. The rate of background mutation in normal human cells is estimated to be in the order of 0.06-1.47 × 10^−9^ per genomic base pair per cell division [32]. Actually, background mutations are not completely random. They are positively correlated with DNA replication and negatively correlated with gene expression level [33]. The genomic regions that are replicated late or the genes that have low expression level usually have high mutation rate. Recently, a novel analytical methodology, MutSigCV, was developed to find the true driver mutations from tons of background mutations [33].

### Functional mutations in hematopoietic stem cells (HSC)

A healthy adult person is believed to produce approximately 10^11^-10^12^new blood cells daily in order to maintain steady state levels in the peripheral circulation [34]. HSCs reside in the medulla of the bone and have the unique ability to give rise to all of the different mature blood cell types and tissues. In normal haematopoiesis, around 1000 HSCs are involved in asymmetric division to facilitate both self-renewal and the generation of differentiated progenies [35]. Given that background mutations could occur during DNA replication and HSC maintain life-long division, it is tempting to consider that some functional mutations that are able to promote tumor initiation could occur in HSCs [36]. TP53 is an important gene to maintain genome stability and prevent malignant transformation [37, 38]. Due to the random accumulation of background mutation, this gene is estimated to be functionally disabled in at least one HSC in 44% of healthy individuals by the age of 50 [39]. Using a droplet digital polymerase chain reaction (ddPCR) assay, it was found that a therapy-related acute myeloid leukemia (t-AML) patient already carried disease-causing TP53 Y220C mutation at a frequency of 0.0027% before exposure to any cytotoxic chemotherapy drugs. Similar phenomenon was observed in another patient, whose bone marrow carried TP53 H179L mutation at a VAF of 0.05% before the initiation of cytotoxic therapy. After 5.5 years of therapy, t-AML was developed and the VAF of TP53 H179L mutation reached 34.7% [39]. Therefore, evidences exist to support the notion that bone marrow could be hit by chance to inactive crucial tumor suppressor genes to facilitate tumor occurrence. It is not known whether these bone marrow cells could finally turn into leukemia stem cell or cancer stemloids (proliferating self-renewing stem cell-like cells) [40].

Besides the linear evolution model during which cells serially acquire somatic alterations that increase tumor fitness, other models in which bursts of somatic mutations and extensive genomic remodeling occur in a relative short period of chronological time has been raised [30, 41–43]. New terms like chromothripsis [42] and chromoplexy [30] have been coined to describe the scenario that massive genomic rearrangements are acquired in a single or a few catastrophic events, which have been recently reviewed in several papers [44–46].

## CONCLUSIONS

Deep sequencing technology has been employed to dissect genome lesions in AML as well as in other tumors. Besides nucleotide alterations such as SNVs and indels, and chromosome alterations, such as CNVs and structural variations (SVs), this technology can also reveal tumor clonal architecture and evolution. It is rather straightforward to understand the potential deleterious effect of SNVs, indels, CNVs, and SVs that lead to amino acid substitution, frame shifting, gene fusion or dysregulated gene expression, how tumor clonal architecture and evolution are inferred is a little bit tortuous. By illustrating the notion of allele frequency, this review demonstrates the principles of converting the information of VAF clusters into tumor clonal structures. Genomic heterogeneity and clonal diversity are the major reason of tumor therapy failure. The clonal architecture revealed by deep sequencing may prove it necessary to use a combination of therapies at the earliest possible time, akin to combination antiretroviral therapy in HIV [47]. Leukemia stem cells could emerge from HSCs or more differentiated progenitors that have obtained appropriate mutations [48, 49]. Deep sequencing is able to reveal tumor clonal architecture. But whether the architecture reflects the heterogeneous nature of LSCs is yet to be determined.

## References

[1] Castro-Giner F, Ratcliffe P, Tomlinson I (2015). The mini-driver model of polygenic cancer evolution. Nature reviews Cancer.

[2] Tabassum DP, Polyak K (2015). Tumorigenesis: it takes a village. Nature reviews Cancer.

[3] Hanahan D, Weinberg RA (2011). Hallmarks of cancer: the next generation. Cell.

[4] Farber E (1984). The multistep nature of cancer development. Cancer research.

[5] Vogelstein B, Kinzler KW (1993). The multistep nature of cancer. Trends in genetics.

[6] Stratton MR, Campbell PJ, Futreal PA (2009). The cancer genome. Nature.

[7] Meyerson M, Gabriel S, Getz G (2010). Advances in understanding cancer genomes through second-generation sequencing. Nature reviews Genetics.

[8] Stratton MR (2011). Exploring the genomes of cancer cells: progress and promise. Science.

[9] Stratton MR (2013). Journeys into the genome of cancer cells. EMBO molecular medicine.

[10] Watson IR, Takahashi K, Futreal PA, Chin L (2013). Emerging patterns of somatic mutations in cancer. Nature reviews Genetics.

[11] Ding L, Ley TJ, Larson DE, Miller CA, Koboldt DC, Welch JS, Ritchey JK, Young MA, Lamprecht T, McLellan MD, McMichael JF, Wallis JW, Lu C, Shen D, Harris CC, Dooling DJ (2012). Clonal evolution in relapsed acute myeloid leukaemia revealed by whole-genome sequencing. Nature.

[12] Falini B, Mecucci C, Tiacci E, Alcalay M, Rosati R, Pasqualucci L, La Starza R, Diverio D, Colombo E, Santucci A, Bigerna B, Pacini R, Pucciarini A, Liso A, Vignetti M, Fazi P (2005). Cytoplasmic nucleophosmin in acute myelogenous leukemia with a normal karyotype. The New England journal of medicine.

[13] King-Underwood L, Renshaw J, Pritchard-Jones K (1996). Mutations in the Wilms' tumor gene WT1 in leukemias. Blood.

[14] Kirito K, Sakoe K, Shinoda D, Takiyama Y, Kaushansky K, Komatsu N (2008). A novel RUNX1 mutation in familial platelet disorder with propensity to develop myeloid malignancies. Haematologica.

[15] Ley TJ, Ding L, Walter MJ, McLellan MD, Lamprecht T, Larson DE, Kandoth C, Payton JE, Baty J, Welch J, Harris CC, Lichti CF, Townsend RR, Fulton RS, Dooling DJ, Koboldt DC (2010). DNMT3A mutations in acute myeloid leukemia. The New England journal of medicine.

[16] Ward PS, Patel J, Wise DR, Abdel-Wahab O, Bennett BD, Coller HA, Cross JR, Fantin VR, Hedvat CV, Perl AE, Rabinowitz JD, Carroll M, Su SM, Sharp KA, Levine RL, Thompson CB (2010). The common feature of leukemia-associated IDH1 and IDH2 mutations is a neomorphic enzyme activity converting alpha-ketoglutarate to 2-hydroxyglutarate. Cancer cell.

[17] Barjesteh van Waalwijk van Doorn-Khosrovani S, Spensberger D, de Knegt Y, Tang M, Lowenberg B, Delwel R (2005). Somatic heterozygous mutations in ETV6 (TEL) and frequent absence of ETV6 protein in acute myeloid leukemia. Oncogene.

[18] Gao J, Erickson P, Gardiner K, Le Beau MM, Diaz MO, Patterson D, Rowley JD, Drabkin HA (1991). Isolation of a yeast artificial chromosome spanning the 8;21 translocation breakpoint t(8;21)(q22;q22. 3) in acute myelogenous leukemia. Proc Natl Acad Sci U S A.

[19] Mardis ER, Ding L, Dooling DJ, Larson DE, McLellan MD, Chen K, Koboldt DC, Fulton RS, Delehaunty KD, McGrath SD, Fulton LA, Locke DP, Magrini VJ, Abbott RM, Vickery TL, Reed JS (2009). Recurring mutations found by sequencing an acute myeloid leukemia genome. N Engl J Med.

[20] Nakao M, Yokota S, Iwai T, Kaneko H, Horiike S, Kashima K, Sonoda Y, Fujimoto T, Misawa S (1996). Internal tandem duplication of the flt3 gene found in acute myeloid leukemia. Leukemia.

[21] Van Vlierberghe P, Patel J, Abdel-Wahab O, Lobry C, Hedvat CV, Balbin M, Nicolas C, Payer AR, Fernandez HF, Tallman MS, Paietta E, Melnick A, Vandenberghe P, Speleman F, Aifantis I, Cools J (2011). PHF6 mutations in adult acute myeloid leukemia. Leukemia.

[22] Ley TJ, Mardis ER, Ding L, Fulton B, McLellan MD, Chen K, Dooling D, Dunford-Shore BH, McGrath S, Hickenbotham M, Cook L, Abbott R, Larson DE, Koboldt DC, Pohl C, Smith S (2008). DNA sequencing of a cytogenetically normal acute myeloid leukaemia genome. Nature.

[23] Cancer Genome Atlas Research N (2013). Genomic and epigenomic landscapes of adult de novo acute myeloid leukemia. The New England journal of medicine.

[24] Walter MJ, Shen D, Ding L, Shao J, Koboldt DC, Chen K, Larson DE, McLellan MD, Dooling D, Abbott R, Fulton R, Magrini V, Schmidt H, Kalicki-Veizer J, O'Laughlin M, Fan X (2012). Clonal architecture of secondary acute myeloid leukemia. The New England journal of medicine.

[25] Engle EK, Fisher DA, Miller CA, McLellan MD, Fulton RS, Moore DM, Wilson RK, Ley TJ, Oh ST (2015). Clonal evolution revealed by whole genome sequencing in a case of primary myelofibrosis transformed to secondary acute myeloid leukemia. Leukemia.

[26] Imielinski M, Berger AH, Hammerman PS, Hernandez B, Pugh TJ, Hodis E, Cho J, Suh J, Capelletti M, Sivachenko A, Sougnez C, Auclair D, Lawrence MS, Stojanov P, Cibulskis K, Choi K (2012). Mapping the hallmarks of lung adenocarcinoma with massively parallel sequencing. Cell.

[27] George J, Lim JS, Jang SJ, Cun Y, Ozretic L, Kong G, Leenders F, Lu X, Fernandez-Cuesta L, Bosco G, Muller C, Dahmen I, Jahchan NS, Park KS, Yang D, Karnezis AN (2015). Comprehensive genomic profiles of small cell lung cancer. Nature.

[28] Bhatia-Gaur R, Donjacour AA, Sciavolino PJ, Kim M, Desai N, Young P, Norton CR, Gridley T, Cardiff RD, Cunha GR, Abate-Shen C, Shen MM (1999). Roles for Nkx3.1 in prostate development and cancer. Genes & development.

[29] Sun C, Dobi A, Mohamed A, Li H, Thangapazham RL, Furusato B, Shaheduzzaman S, Tan SH, Vaidyanathan G, Whitman E, Hawksworth DJ, Chen Y, Nau M, Patel V, Vahey M, Gutkind JS (2008). TMPRSS2-ERG fusion, a common genomic alteration in prostate cancer activates C-MYC and abrogates prostate epithelial differentiation. Oncogene.

[30] Baca SC, Prandi D, Lawrence MS, Mosquera JM, Romanel A, Drier Y, Park K, Kitabayashi N, MacDonald TY, Ghandi M, Van Allen E, Kryukov GV, Sboner A, Theurillat JP, Soong TD, Nickerson E (2013). Punctuated evolution of prostate cancer genomes. Cell.

[31] Welch JS, Ley TJ, Link DC, Miller CA, Larson DE, Koboldt DC, Wartman LD, Lamprecht TL, Liu F, Xia J, Kandoth C, Fulton RS, McLellan MD, Dooling DJ, Wallis JW, Chen K (2012). The origin and evolution of mutations in acute myeloid leukemia. Cell.

[32] Lynch M (2010). Rate, molecular spectrum, and consequences of human mutation. Proceedings of the National Academy of Sciences of the United States of America.

[33] Lawrence MS, Stojanov P, Polak P, Kryukov GV, Cibulskis K, Sivachenko A, Carter SL, Stewart C, Mermel CH, Roberts SA, Kiezun A, Hammerman PS, McKenna A, Drier Y, Zou L, Ramos AH (2013). Mutational heterogeneity in cancer and the search for new cancer-associated genes. Nature.

[34] Beerman I, Maloney WJ, Weissmann IL, Rossi DJ (2010). Stem cells and the aging hematopoietic system. Current opinion in immunology.

[35] Catlin SN, Busque L, Gale RE, Guttorp P, Abkowitz JL (2011). The replication rate of human hematopoietic stem cells *in vivo*. Blood.

[36] Grove CS, Vassiliou GS (2014). Acute myeloid leukaemia: a paradigm for the clonal evolution of cancer?. Disease models & mechanisms.

[37] Kern SE, Kinzler KW, Bruskin A, Jarosz D, Friedman P, Prives C, Vogelstein B (1991). Identification of p53 as a sequence-specific DNA-binding protein. Science.

[38] Surget S, Khoury MP, Bourdon JC (2013). Uncovering the role of p53 splice variants in human malignancy: a clinical perspective. OncoTargets and therapy.

[39] Wong TN, Ramsingh G, Young AL, Miller CA, Touma W, Welch JS, Lamprecht TL, Shen D, Hundal J, Fulton RS, Heath S, Baty JD, Klco JM, Ding L, Mardis ER, Westervelt P (2015). Role of TP53 mutations in the origin and evolution of therapy-related acute myeloid leukaemia. Nature.

[40] Blagosklonny MV (2007). Cancer stem cell and cancer stemloids: from biology to therapy. Cancer biology & therapy.

[41] Berger MF, Lawrence MS, Demichelis F, Drier Y, Cibulskis K, Sivachenko AY, Sboner A, Esgueva R, Pflueger D, Sougnez C, Onofrio R, Carter SL, Park K, Habegger L, Ambrogio L, Fennell T (2011). The genomic complexity of primary human prostate cancer. Nature.

[42] Stephens PJ, Greenman CD, Fu BY, Yang FT, Bignell GR, Mudie LJ, Pleasance ED, Lau KW, Beare D, Stebbings LA, McLaren S, Lin ML, McBride DJ, Varela I, Nik-Zainal S, Leroy C (2011). Massive Genomic Rearrangement Acquired in a Single Catastrophic Event during Cancer Development. Cell.

[43] Crasta K, Ganem NJ, Dagher R, Lantermann AB, Ivanova EV, Pan Y, Nezi L, Protopopov A, Chowdhury D, Pellman D (2012). DNA breaks and chromosome pulverization from errors in mitosis. Nature.

[44] Leibowitz ML, Zhang CZ, Pellman D (2015). Chromothripsis: A New Mechanism for Rapid Karyotype Evolution. Annual review of genetics.

[45] Willis NA, Rass E, Scully R (2015). Deciphering the Code of the Cancer Genome: Mechanisms of Chromosome Rearrangement. Trends in cancer.

[46] Zhang X, Deng X, Zhang Y, Li Z (2015). Novel patterns of cancer genome evolution. Oncology and Translational Medicine.

[47] Goldie JH, Coldman AJ (1984). The genetic origin of drug resistance in neoplasms: implications for systemic therapy. Cancer research.

[48] Jordan CT (2007). The leukemic stem cell. Best practice & research Clinical haematology.

[49] Schepers K, Campbell TB, Passegue E (2015). Normal and leukemic stem cell niches: insights and therapeutic opportunities. Cell stem cell.

